# Upregulation of EMMPRIN (OX47) in Rat Dorsal Root Ganglion Contributes to the Development of Mechanical Allodynia after Nerve Injury

**DOI:** 10.1155/2015/249756

**Published:** 2015-11-30

**Authors:** Qun Wang, Yanyuan Sun, Yingna Ren, Yandong Gao, Li Tian, Yang Liu, Yanan Pu, Xingchun Gou, Yanke Chen, Yan Lu

**Affiliations:** ^1^Department of Anesthesiology and Pain Management, Xijing Hospital, Fourth Military Medical University, Xi'an 710032, China; ^2^Department of Anesthesiology, Yulin First Hospital, Yulin 710021, China; ^3^Institute of Basic Medical Science, Xi'an Medical University, Xi'an 710021, China; ^4^Experiment Center of Biomedical Research, School of Medicine, Xi'an Jiaotong University, Xi'an 710061, China

## Abstract

Matrix metalloproteinases (MMPs) are widely implicated in inflammation and tissue remodeling associated with various neurodegenerative diseases and play an important role in nociception and allodynia. Extracellular Matrix Metalloproteinase Inducer (EMMPRIN) plays a key regulatory role for MMP activities. However, the role of EMMPRIN in the development of neuropathic pain is not clear. Western blotting, real-time quantitative RT-PCR (qRT-PCR), and immunofluorescence were performed to determine the changes of messenger RNA and protein of EMMPRIN/OX47 and their cellular localization in the rat dorsal root ganglion (DRG) after nerve injury. Paw withdrawal threshold test was examined to evaluate the pain behavior in spinal nerve ligation (SNL) model. The lentivirus containing OX47 shRNA was injected into the DRG one day before SNL. The expression level of both mRNA and protein of OX47 was markedly upregulated in ipsilateral DRG after SNL. OX47 was mainly expressed in the extracellular matrix of DRG. Administration of shRNA targeted against OX47 in vivo remarkably attenuated mechanical allodynia induced by SNL. In conclusion, peripheral nerve injury induced upregulation of OX47 in the extracellular matrix of DRG. RNA interference against OX47 significantly suppressed the expression of OX47 mRNA and the development of mechanical allodynia. The altered expression of OX47 may contribute to the development of neuropathic pain after nerve injury.

## 1. Introduction

Neuropathic pain caused by a lesion or disease of the somatosensory system is refractory to routine analgesic measures [1, 2]. Following nerve injury, the sensory nervous system undergoes maladaptive changes that result in neuronal hyperexcitability [3–5]. The spinal dorsal horn is a relay station in which sensory information from dorsal root ganglia (DRG) is received, integrated, and relayed to several brain regions. Multiple alterations distributed widely across the peripheral and central nervous system contribute to the development of neuropathic pain. The peripheral nervous system is subject to damage, and the alterations are evident in the DRG. Despite the fact that intensive research activity is focused on the changes of ion channels, growth factors, cytokines, and glia cells in the DRG [5], the most inchoate alterations after nerve injury are not fully identified.

Matrix metalloproteinases (MMPs) are a family of zinc-dependent endopeptidases that play crucial roles in a wide range of proteolytic processes. More than 20 members of the family were reported, such as Collagenase-1 (MMP-1), Stromelysin-1 (MMP-3), Matrilysin (MMP-7), Gelatinase A (MMP-2), Gelatinase B (MMP-9), and MT1-MMP (MMP-14) [6, 7]. Earlier studies mainly shed light on the functions of MMPs in the physiological state. Recent studies suggested that MMPs are widely implicated in inflammation and tissue remodeling associated with various neurodegenerative diseases through the cleavage of the extracellular matrix and enhancement of cytokines, chemokines, growth factors, cell surface receptors, and cell adhesion molecules [5, 6]. Meanwhile, they are also involved in supporting regeneration and vascular remodeling processes [7–9]. When the nervous system is injured, transcription and synthesis of MMPs in several cell types will increase to promote local repair, remyelination, regeneration, and even angiogenesis [10–13]. Moreover, recent studies demonstrated that MMPs also play crucial roles in nociception and hyperalgesia [10, 14], especially in the neuropathic pain and migraine [10, 15]. MMP-9 and MMP-2 were found to be involved in the development of neuropathic pain [16].

Extracellular Matrix Metalloproteinase Inducer (EMMPRIN) plays a key regulatory role in several MMPs activities [17–19]. CD147 (human), OX47 (rat), basigin, M6 antigen, neurothelin, HT7, and gp42 are different names for EMMPRIN in different species [17–20]. Numerous studies have shown that EMMPRIN display a remarkable repertoire of biological functions, including cell growth and migration, tissue regeneration, and cell differentiation and adhesion. Excessive expression of EMMPRIN was demonstrated to increase the invasiveness of tumor cells and play a role in the pathophysiology of various disease processes [21–24], such as atherosclerosis [25], acute myocardial infarction [26, 27], and transient [28] and permanent focal cerebral ischemia [29]. In vivo study showed that altered MMP expressions of tumor stromal fibroblasts were closely correlated with the expression level of CD147 [30–32]. Relevant studies manifested that fibroblasts transfected with restructuring CD147 adenovirus vector upregulated the expressions of MMP-1 and MMP-3 [33].

The role of EMMPRIN in the development of neuropathic pain induced by nerve injury is not clear. The present study examined the expression changes of OX47 in the DRG and spinal dorsal horn in neuropathic pain condition induced by peripheral nerve injury.

## 2. Materials and Methods

### 2.1. Animals

Male Sprague-Dawley rats (200–220 g), purchased from Animal Center of Fourth Military Medical University, were housed in groups of six under the constant temperature (25 ± 1)°C and 12 h light/dark cycle with free access to food and water. Behavioral tests were conducted by an observer blind to the behavioral analysis and drug treatments. All the operating procedures, in accordance with ethical guidelines, were approved by the Animal Care Committee.

### 2.2. Spinal Nerve Ligation (SNL) Model

All animals were deeply anesthetized with sodium pentobarbital (40 mg/kg) by intraperitoneal injection. Then the L5 spinal nerve ligation (SNL) surgery was conducted as previously described [34]. Briefly, the L5 spinal nerve was isolated through the removal of spinal transverse process. Subsequently, the L5 nerve was tightly ligated with 5-0 silk thread twice in 0.3 cm interval. Then, the incision was sutured layer by layer. After the surgery, all animals were transmitted to feeding room, where they were closely monitored to recover from the operation. Control surgery was subjected to the same procedure except for the ligation of L5 spinal nerve.

### 2.3. Behavioral Analysis

All behavioral tests were performed by a blinded observer. Rats were placed individually in cages (30 cm × 30 cm × 30 cm) with a wire mesh bottom (1 cm × 1 cm × 1 cm) and fully habituated three times prior to the assessment of paw withdrawal mechanical threshold (PWMT) with a frequency of one hour every day. One day before the SNL surgery, the baseline of PWMT was measured. One day after surgery, the PWMT was measured again to exclude the unsuccessful model (motor dysfunction or pain threshold value did not decrease obviously) followed by measurement at different postoperative days (1 d, 3 d, 7 d, 14 d, and 21 d). After acclimatization (30 min), mechanical nociceptive thresholds were determined by paw withdrawal to stimulation of the glabrous surface of the paw. Calibrated von Frey filaments (Stoelting, 1, 1.5, 2, 4, 6, 8.3, 11.1, 16.5, and 26 g) were applied with enough force to cause buckling of the filament. If rats appeared to shake the foot, lick foot, or show withdrawal behavior within 5 s, then it was defined as positive reaction. At the same force, the measurement was repeated 5 times at intervals of 5 minutes. Once more than sixty percent of measurement appeared as positive reaction, the minimal force was determined as the paw withdrawal mechanical threshold.

### 2.4. Western Blotting

Rats were transcardially perfused with 0.1 M PBS after deep anesthesia with sodium pentobarbital. The L5 DRGs and spinal cord segments were then rapidly removed, followed by being homogenized in a RIPA lysis buffer (Beyotime Inc., Nantong, China) containing a proteinase inhibitors phenylmethanesulfonyl fluoride (PMSF, 1 mM) (Beyotime Inc., Nantong, China). Protein concentrations were measured by the BCA Assay (Pierce Biotechnology) and 15 *μ*g proteins were loaded, separated on a 12% SDS-PAGE, and transferred to polyvinylidene difluoride membranes (PVDF). The PVDF membranes were subsequently immunoblotted with the appropriate primary antibodies including mouse monoclonal antibody to OX47 (1 : 500, Santa Cruz Biotech, US) and anti-rat actin mouse monoclonal antibody (20 *μ*g/mL, Chemicon International, Inc.). After being extensively washed, the membranes were incubated with a horseradish peroxidase-conjugated goat anti-mouse secondary antibody (Zhongshanqinqiao, Beijing, China). Signals were detected by an ECL kit (Pierce Biotechnology) according to the manufacturer's instructions.

### 2.5. Real-Time Quantitative RT-PCR (qRT-PCR)

Total RNA were extracted from tissues by Trizol (Invitrogen, US). cDNA was synthesized by superscript first strand synthesis kit (Invitrogen, US) according to the manufacturer's standard protocol. Then, OX47 and glyceraldehyde-3-phosphate dehydrogenase (GAPDH) mRNA expression levels were measured by Mini-Opticon real-time PCR detection system (Bio-Rad, Hercules, CA, USA) in SYBR Green master mix (Takara, Otsu, Japan) according to a standard protocol. Finally, all data were analyzed by Opticon Monitor software (version 3.1; Bio-Rad). All primers were synthesized by the Shanghai Sangon Biological Engineering Technology and Services Co. Ltd. (Shanghai, China). The sequences of PCR primers are listed:

OX47:

 5′-GTTTGTGAAGCTGATCTGCAAG-3′
 5′-ACAGCTCAGGCGTGGATATAAT-3′

GAPDH:

 5′-GGCAAGTTCAATGGCACAGT-3′
 5′-TGGTGAAGACGCCAGTAGACTC-3′

### 2.6. Immunofluorescence Chemistry

The rats were deeply anesthetized with sodium pentobarbital (40 mg/kg) and perfused through the ascending aorta with saline followed by 4% paraformaldehyde. The L5 spinal cord and dorsal root ganglion were postfixed with the same fixative for 2 h and then cryoprotected in a solution of 20% sucrose in 1% phosphate buffer (pH 7.4) overnight at room temperature. The serial coronal sections (40 *μ*m) were cut by a cryostat microtome (CM1900, Leica, Germany). In double labeling experiments, all sections were blocked with 2% goat serum and incubated overnight at 4°C with mouse monoclonal antibody to OX47 (1 : 500, Santa Cruz Biotech, US), polyclonal rabbit anti-glial fibrillary acidic protein (1 : 1000, DAKO, US), anti-Collagen IV (1 : 1000, Sigma, US), and anti-Laminin (1 : 1000, Sigma, US). Subsequently, the slices were washed with 0.01% PBS three times and incubated with the respective Cy3-conjugated or FITC-conjugated (1 : 1000, Sigma, US) secondary antibody for 30 minutes in the dark at room temperature. Negative controls were conducted by omitting the primary antibodies during the immunostaining. After immunostaining, sections were mounted with 50% glycerol and covered with coverslips. Eventually, the sections were observed by using a laser scanning confocal microscope (Olympus FV1000, Tokyo, Japan). Images were adjusted by the FV10-ASW 3.1 Viewer software.

### 2.7. Construction of shRNA Lentiviral Vector

The lentiviral vector pMAGic 4.1 (SunBio Inc., Shanghai, China) was used to generate short hairpin RNA (shRNA) specific for rat EMMPRIN (OX-47). Four different regions of OX47 mRNA (gene ID NM 001109882) were selected as the RNAi target sites, namely, 394–412 bp, 756–774 bp, 900–918 bp, and 1206–1220 bp. Another pair of oligonucleotides (designated as shRNA-control) encoding a nonspecific shRNA, a negative control, was also synthesized. These oligonucleotides were annealed and subcloned into the Age I and EcoR I restriction endonuclease sites of pMAGic 4.1 lentiviral vector (SunBio, Shanghai, China), which included the green fluorescent protein (GFP) tag. All of the constructed vectors were confirmed by DNA sequencing.

HEK293 cells and MADB106 mammary adenocarcinoma cells were cultured in medium containing RPMI 1640 (Gibco, BRL Life Technologies, Karlsruhe, Germany) with 10% foetal bovine serum (FBS, Gibco) at 37°C under a mixture of 95% air and 5% CO2. HEK293 cells were cotransfected with these lentiviral vectors together with packaged plasmid pMD2.G (Addgene plasmid # 12259) and psPAX2 (Addgene plasmid # 12260) by Lipofectamine 2000 (Invitrogen, Carlsbad, CA, USA) according to the manufacturer's instructions. Forty-eight hours post transfection, harvested MADB106 cells were then infected with 1 mL of viral stock containing 5 *μ*g/mL polybrene and enhanced infection solution (Eni.S) for 48 h. Then this medium was replaced by normal culture medium. The interference efficiency was verified by quantitative qRT-PCR. The shRNA oligonucleotides with maximum effect (75%) of silencing for OX47 were listed below:

 5′-CCGGGGACACAGGCACTTATGAATTCAAGAGATTCATAAGTGCCTGTGTCCTTTTTTg -3′
 5′-AATTCAAAAAAGGACACAGGCACTTATGAATCTCTTGAATTCATAAGTGCCTGTGTCC-3′

### 2.8. Injection of Lentivirions

Lentivirions expressing either GFP or shRNA against OX47 were injected into DRG in vivo using a published method modified version [35]. Briefly, lentiviral preparations (1 × 10^9^ transfection units per mL) were diluted with 20% mannitol in a ratio of 1 : 1 and injected unilaterally into L5 DRGs (6 *μ*L per DRG, or 3 × 10^6^ transfection units per DRG) in deeply anesthetized condition. At second day after viral infection, rats were subjected to SNL as described above. Mechanical hyperalgesia was measured by a von Frey monofilament. Rats were sacrificed on 7–22 days after SNL; then the L4-L5 DRGs were rapidly isolated and subjected to Western blot analysis for OX47 and *β*-actin.

### 2.9. Statistics

Student's *t*-test or analysis of variance (ANOVA) for random measures was performed followed by post hoc Fisher's test to determine statistically significant differences. Statistical significance was taken at *P* < 0.05. All data were presented as mean ± SEM.

## 3. Results

### 3.1. The Expression of OX47 Was Exclusively Upregulated in DRG after SNL

**Figure 1:**
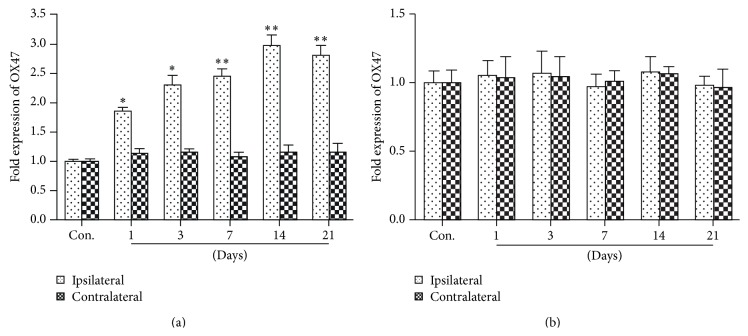
Upregulation of OX47 mRNA in the DRG after spinal nerve ligation (SNL). (a) The expression level of OX47 mRNA in DRGs of control and SNL group. mRNA levels were determined by real-time quantitative RT-PCR (qRT-PCR). Nerve injury induced a significant upregulation of OX47 mRNA expression at ipsilateral DRG. *n* = 8 per group. ^*∗*^ *P* < 0.05, ^*∗∗*^ *P* < 0.01. (b) The expression level of OX47 mRNA in spinal cord of control and SNL group. There are no significant differences between the ipsilateral and contralateral spinal cord. *n* = 8 per group. *P* > 0.05.

**Figure 2:**
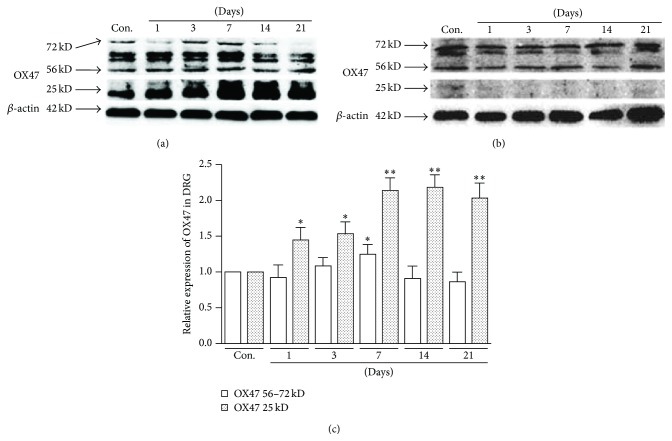
Upregulation of OX47 protein in the DRG after spinal nerve ligation (SNL). (a) The expression level of OX47 protein in DRGs of control and SNL group. Protein levels were determined by Western blotting. Note that the nonglycosylated OX47 (25 kd) protein expression was increased from the first day after SNL. (b) The expression level of OX47 protein in spinal cord of control and SNL group. Note that only glycosylated protein was detected with low level. (c) Statistical data indicated that the increased expression of OX47 protein in DRG occurred from the first day after SNL, reached the peak at the fourteenth day, and was maintained at a relatively stable level for at least 21 days. *n* = 6 per group. ^*∗*^ *P* < 0.05, ^*∗∗*^ *P* < 0.01.

qRT-PCR and Western blot were used to assess the changes of OX47 expression in DRG and spinal cord after SNL. OX47 molecules include glycosylated (MW 56–72 kD) and nonglycosylated (MW 25 kD) forms. The expression level of both mRNA (Figure 1(a)) and protein (Figures 2(a) and 2(c)) of OX47 in the ipsilateral (SNL) L5 DRG was significantly increased compared to the contralateral side. Particularly, the nonglycosylated OX47 with 25 kD was exceptionally enhanced. The increased expression of mRNA and protein occurred from the first day after SNL, reached the peak at the fourteenth day, and was maintained at a relatively stable level for at least 21 days (Figures 1(a) and 2(c)). However, the expression level of both mRNA (Figure 1(b)) and protein (Figure 2(b)) of OX47 in the spinal dorsal horn showed no significant difference between the ipsilateral and contralateral sides. In line with the mRNA expression, only the glycosylated rather than nonglycosylated OX47 was detected with low level expression in Western blotting (Figure 2(b)). Taken together, these data indicated that peripheral nerve injury significantly augmented the expression of EMMPRIN in spinal DRG.

### 3.2. OX47 Was Mainly Expressed in the Extracellular Matrix of DRG

**Figure 3:**
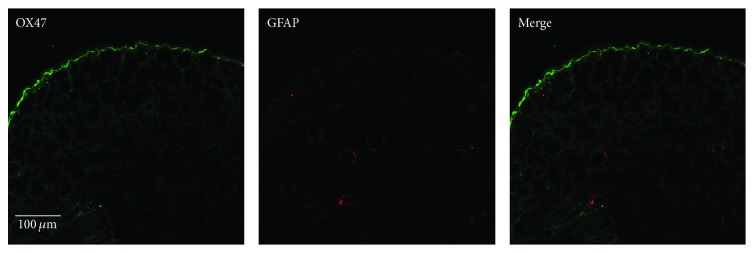
Double-labeled immunostaining of OX47 with GFAP in naïve DRG. Representative confocal microscopy shows the immunoreactivity (green) for OX47 and GFAP (red).

**Figure 4:**
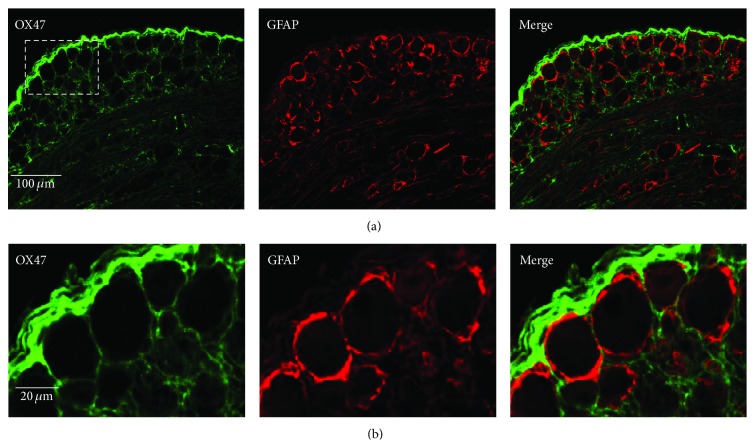
Double-labeled immunostaining of OX47 with GFAP in DRG 3 days after SNL. (a) Representative confocal microscopy shows that OX47 immunoreactivity was mainly detected in the extracellular matrix around DRG neurons and partially overlapped with flattened SGCs positive for GFAP. (b) Higher magnification of selected area in (a).

To further characterize the cellular localization of OX47 in DRG, we carried out double-labeled immunostaining of OX47 with glial fibrillary acidic protein (GFAP), a marker of astrocyte (satellite glial cell in DRG). In the naïve rat, both OX47 and GFAP were expressed at a low level (Figure 3). Three days after SNL, the activated satellite glial cells (SGCs) encircled and surrounded large-, medium-, and small-sized DRG neurons (Figure 4). OX47 immunoreactivity was mainly detected in the extracellular matrix around DRG neurons and partially overlapped with flattened SGCs positive for GFAP (Figure 4). Clearly, OX47 immunoreactivity was not colocalized with DRG neurons.

**Figure 5:**
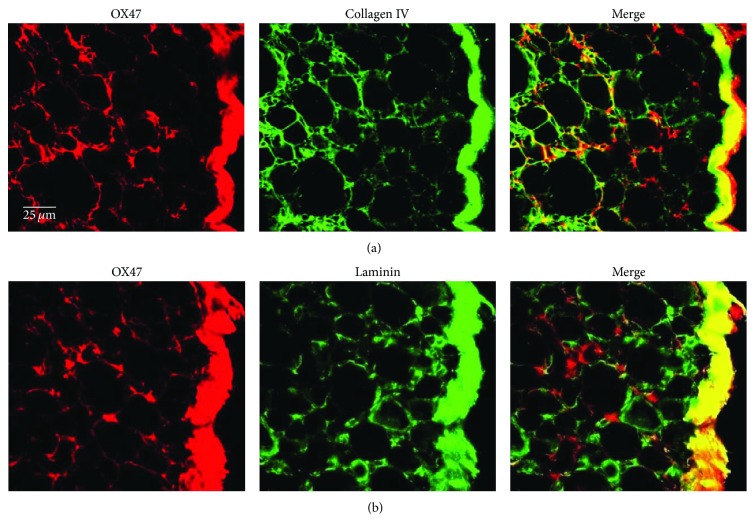
Double-labeled immunostaining of OX47 with Collagen IV and Laminin in DRG. (a) OX47 positive labeling was colocalized with Collagen IV in DRG's capsule and extracellular matrix. (b) OX47 was mainly colocalized with Laminin in DRG's capsule.

Collagen IV and Laminin are major components of the extracellular matrix. We next performed double labeling of OX47 with Collagen IV and Laminin. Indeed, OX47 immunoreactivity was overlapped with Collagen IV in DRG's capsule and extracellular matrix (Figure 5(a)), while the Laminin immunoreactivity was mainly colocalized with OX47 in DRG's capsule (Figure 5(b)). Overall, the colocalization with Collagen IV, SGCs, and Laminin suggested that OX47 was mainly expressed in the extracellular matrix of DRG.

### 3.3. Pretreatment with OX47 shRNA Markedly Attenuated the Development of Mechanical Allodynia

**Figure 6:**
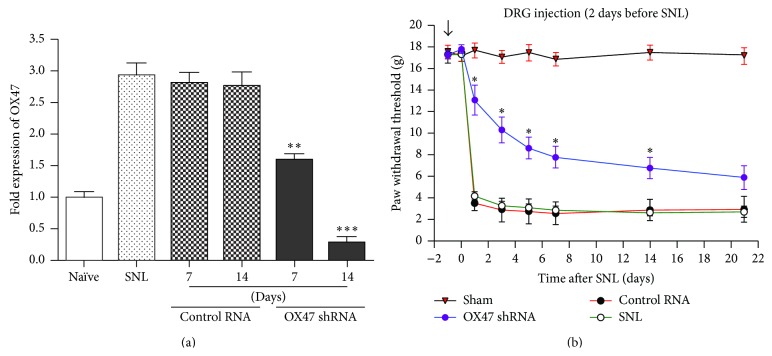
DRG injection of OX47 shRNA markedly attenuated the development of mechanical allodynia. (a) OX47 mRNA expression in DRG of control RNA and OX47 shRNA group. Data show that OX47 shRNA obviously decreased the mRNA expression in the 7 d and 14 d after SNL. *n* = 5 per group. ^*∗∗*^ *P* < 0.01, ^*∗∗∗*^ *P* < 0.001. (b) Increased paw withdrawal threshold after OX47 shRNA injection. Note that mechanical allodynia was remarkably attenuated in rats infected with lentivirus containing shRNA of OX47. *n* = 7 per time point. ^*∗*^ *P* < 0.05 compared to nonsilencing control shRNA (nonspecific scrambled shRNA).

To further shed light on the functional role of OX47 in neuropathic pain condition, we constructed a shRNA lentiviral vector and completed packaging the lentivirus. Seven days after injection of the shRNA into DRG, the expression of OX47 mRNA in the DRG was significantly suppressed (Figure 6(a)). In contrast to the OX47 shRNA, the control RNA did not significantly alter the OX47 mRNA expression. We next assessed the effect of OX47 shRNA in the SNL model. Pretreatment of OX47 shRNA significantly increased the paw withdrawal threshold as compared with control RNA-treated animals and thus attenuated the mechanical allodynia as expected (Figure 6(b)). These data indicated that pretreatment with OX47 shRNA markedly attenuated the development of mechanical allodynia after nerve injury.

## 4. Discussion

The present study provided evidence that upregulation of EMMPRIN in the dorsal root ganglion was involved in the development of mechanical allodynia after nerve injury. Firstly, the expression of EMMPRIN mRNA and protein was apparently increased in DRG after SNL, which is consistent with the notion that DRG neurons undergo significant plastic changes in neuropathic pain conditions [36]. Secondly, EMMPRIN was colocalized with Collagen IV, Laminin-1, and SGCs, indicating that EMMPRIN was mainly expressed in the extracellular matrix of DRG. DRG neurons were surrounded by the flattened SGCs and basal laminae of the extracellular matrix [37, 38]. The basal lamina consists of various types of molecules including type IV Collagen, Laminins, entactin/nidogen, and heparan sulfate proteoglycans [39–41]. SGCs are linked to each other by adherent gap junctions [37] and contain several ion channels, receptors, and adhesion molecules [38]. Following peripheral nerve injury, SGCs underwent changes in cell number, structure, and function [38, 42–45]. Several reports demonstrated that GFAP expression was upregulated in SGCs after SNL [46–48]. The MMP-2 was also upregulated in SGCs after SNL [16]. The upregulation of EMMPRIN in extracellular matrix suggested that nerve injury induced significant changes of molecular content in extracellular matrix surrounding the neuron-SGC units. Finally, administration of the EMMPRIN shRNA significantly suppressed mechanical allodynia induced by spinal nerve injury.

It was well accepted that the “Neuron-Glia-Neuron” positive feedback loop leads to the generation and maintenance of neuropathic pain. After nerve injury, proinflammatory cytokines such as IL-6, IL-1*β*, and TNF-*α* were released from activated glial cells in DRG, whereby these factors led to peripheral sensitization by enhancing excitability of DRG neurons [16, 49–53]. The present study suggested that the extracellular matrix may be also involved in the development of neuropathic pain; that is, the formation of “Extracellular Matrix-Glia-Neuron” interactive network may be involved in the development of neuropathic pain. EMMPRIN, the crucial component of extracellular matrix within DRG, may potentially become a novel target for the treatment of neuropathic pain. Inhibition of EMMPRIN may provide a hopeful therapeutic approach for curing neuropathic pain.

In conclusion, peripheral nerve injury induced upregulation of OX47 in the extracellular matrix of DRG. RNA interference against OX47 significantly suppressed the expression of OX47 mRNA and the development of mechanical allodynia. The altered expression of OX47 may contribute to the development of neuropathic pain after nerve injury. Further research is needed to explore which signal pathway EMMPRIN mainly affects during the development of neuropathic pain.
